# An effective inequity reduction intervention: evaluating what made it work

**DOI:** 10.1186/1472-6963-14-S2-P115

**Published:** 2014-07-07

**Authors:** Sivan Spitzer Shohat, Efrat Shadmi, Margalit Goldfracht, Calanit Kay, Ran D Balicer

**Affiliations:** 1 Faculty of Social Welfare and Health Sciences, University of Haifa, Haifa, Israel; 2 Clalit Health Services, Tel-Aviv, Israel

## Background

Recent healthcare literature points to the need to investigate how to achieve inequity reduction. We studied the implementation of an organizational-wide inequity reduction intervention in Israel’s largest healthcare provider and insurer, Clalit Health Services during 2009-2012. The intervention focused on reducing health and healthcare gaps between 55 target clinics (major clinics serving predominantly minority and socioeconomically deprived populations) and all other 126 major primary care clinics. The intervention focused on inequity reduction in a composite weighted score of seven indicators: attainment of diabetes, blood pressure, and lipid control; lack of anemia in infants; and performance of mammography, occult blood tests, and influenza vaccinations.

## Materials and methods

A mixed-methods qualitative-quantitative design assessed implementation in 26 of the 55 target clinics. We assessed intra-organizational ties using social network analysis and perceived team effectiveness (using Shortell’s questionnaire). 108 semi-structured interviews were conducted with clinics’ team members (a physician, nurse, administrator and pharmacist) and their respective managerial units. We mapped the types of interventions using an adaptation of a tool designed to map interventions according to the Chronic Care Model. The relationships between network characteristics, perceived team-effectiveness, type and scope of interventions, and improvement in the composite quality score were assessed.

## Results

At baseline, the composite weighted score for target clinics was 56.8, compared to 63.4 for non-intervention clinics, and at the three-year follow-up 66.7% of this gap was reduced. Among target clinics, those with high intra-network cohesion and intensive relationships with sub-regional management had high ratings on the perceived team effectiveness scale (rs=0.406, p<0.05; rs=0.464, p<0.05). Interventions focused on the organization of care i.e., improvement of teamwork, were found to be positively correlated with improvement in the composite score (rs=0.393, p<0.05). This finding was supported by qualitative data indicating teamwork as the factor attributed most to attainment of success. Furthermore, interventions tailored to community needs, such as work with religious leaders to improve immunizations or nurse-led ethnically adapted cooking classes for diabetic patients, were also found to be positively correlated with improvement in the composite measure (rs=0.449, p<0.05). Conversely, clinics that mainly focused on patient education in specific disease areas (such as diabetes control) did not achieve significant improvement in overall quality.

## Conclusions

This study shows that interventions focused on the organization of care as well as community linkages are correlated with favorable outcomes in care quality and equity within a comprehensive organization-wide inequity reduction program.

